# Computerized cognitive behavioural therapy for gender minority adolescents: Analysis of the real-world implementation of SPARX in New Zealand

**DOI:** 10.1177/0004867420976846

**Published:** 2020-12-08

**Authors:** Mathijs FG Lucassen, Karolina Stasiak, Theresa Fleming, Christopher Frampton, Yael Perry, Matthew Shepherd, Sally N Merry

**Affiliations:** 1 Department of Health and Social Care, Faculty of Wellbeing, Education, and Language Studies, The Open University, Milton Keynes, UK; 2 Department of Psychological Medicine, Faculty of Medical and Health Sciences, The University of Auckland, Auckland, New Zealand; 3 Department of Psychological Medicine, The University of Auckland, Auckland, New Zealand; 4 School of Health, Victoria University of Wellington, Wellington, New Zealand; 5 Department of Psychological Medicine, University of Otago, Christchurch, New Zealand; 6 Telethon Kids Institute, University of Western Australia, Perth, Australia; 7 School of Psychology, Albany Campus, Auckland, New Zealand

**Keywords:** Transgender, adolescent, depression, cognitive behavioural therapy, LGBT

## Abstract

**Objective:**

SPARX is a form of computerized cognitive behavioural therapy in serious game format funded via the Ministry of Health to be freely available in New Zealand. At registration users identify themselves as male, female, transgender or intersex. We aimed to establish whether adolescent transgender users of SPARX, compared to adolescent male and female users, were more likely to have high mental health needs at baseline and were more likely to complete SPARX. We also sought to determine changes in transgender adolescents’ depressive symptoms after using SPARX.

**Methods:**

Quantitative analysis of 5 years of usage data from the nation-wide delivery of SPARX in New Zealand.

**Results:**

There were 9079 adolescents who completed the registration process and used SPARX, 2.3% (*n* = 207) identified as transgender. The majority of transgender registrants (69.0%) completing a baseline Patient Health Questionnaire – modified for Adolescents were categorized as having high mental health needs, significantly more so than male and female registrants (*p* < 0.001). Over half of all SPARX registrants completed the first module of the program, with subsequently lower proportions of transgender registrants completing Module 4 (*p* = 0.005) and Module 7 (i.e. the last module, *p* = 0.048). Of those registrants completing a baseline and subsequent Patient Health Questionnaire – modified for Adolescents, both male (*n* = 247) and female (*n* = 630) registrants, on average, had improvements in their scores (2.68 and 3.15, respectively), whereas transgender registrants (*n* = 14) did not (−0.43) (*p* = 0.048).

**Conclusion:**

This is the first study describing the impact of an e-therapy on transgender young people. The analysis of data from this free self-help intervention suggests that transgender adolescents seeking treatment for depression have particularly high mental health needs, and an existing well tested tool may be less effective for them than it is for others. Taken together the results appear to suggest targeted efforts may be required for transgender adolescents.

## Introduction

Research has consistently highlighted that sexual and gender minority (e.g. lesbian, gay, bisexual and transgender/LGBT) adolescents frequently experience harmful social environments (Hillier et al., 2010; Lucassen et al., 2015a) and depression (Lucassen et al., 2017; Marshal et al., 2011; Reisner et al., 2016). Transgender (trans) and other gender minority adolescents are those young people whose gender identity and/or gender expression contrasts with the norms associated with the sex they were assigned at birth. Trans adolescents are thought to be at an especially high risk of mistreatment which is associated with elevated rates of compromised mental health (Reisner et al., 2016; Strauss et al., 2020a, 2020b). To date, studies in the wider adolescent LGBT health field have been dominated by work that has tended to focus on sexual minority (e.g. lesbian, gay and bisexual) adolescents, with gender minority (e.g. transgender) adolescents infrequently the focus. If included in studies, despite their greater need, gender minority adolescents are often grouped together with sexual minority adolescents, usually resulting in an overall LGBT category (Institute of Medicine, 2011). Moreover, until recently, where research was conducted separately with gender minority people, the studies were primarily conducted with adults. For instance, Reisner et al. (2016) noted ‘a dearth’ of research about transgender young people in their review of the health needs of transgender populations, where only 15 (out of 116) studies published between 2008 and 2014 were conducted with transgender children, adolescents and young people. Their review was inclusive of all quantitative data in relation to disease burden in transgender people of any age (Reisner et al., 2016). Even less has been reported on the experiences of gender minority adolescents which draws on population-based data, making establishing accurate prevalence estimates in relation to mental health problems challenging. For example, a systematic review and meta-analysis of suicide attempts among LGBT youth, based on population-based data, identified only one study where the results for transgender adolescents were presented as a separate group (Di Giacomo et al., 2018). In this particular study (i.e. Clark et al., 2014), transgender adolescents, as cited in Di Giacomo et al.’s (2018) meta-analysis, had an odds ratio of 5.9 (95% confidence intervals [95% CI] = [3.5, 9.8]) in regard to suicide attempts compared to their peers who were not transgender (i.e. are cisgender). This same study, which was nationally representative of high school students in New Zealand, reported that 41.3% of transgender adolescents had clinically significant depressive symptoms, compared to 11.8% of cisgender adolescents (odds ratio 5.7, 95% CI = [3.6, 9.2]) (Clark et al., 2014). However, research on adolescent gender minority individuals and their mental health has expanded considerably in recent years. For example, work published since Reisner et al.’s (2016) review has suggested higher rates of mental health problems not just among transgender adolescents when compared to cisgender adolescents, but also when compared to transgender people in older age groups (James et al., 2016; Tan et al., 2020).

Given that gender minority adolescents are an under-served population in terms of their mental health needs further efforts have been recommended in relation to refining, researching and delivering evidence-based psychotherapies to this unique population (Austin and Craig, 2015). At present only a very small collection of interventions are focused on reducing mental health problems among gender as well as sexual minority adolescents, with the emphasis in treatment so far being on sexual minority adolescents (Coulter et al., 2019). In particular, a systematic review (to January 2019) found only nine interventions in the peer-reviewed literature designed to reduce substance use, mental health problems or victimization in gender and sexual minority adolescents (Coulter et al., 2019). Only three of these were gender minority–specific interventions, and these all examined transition-related gender-affirming care (e.g. the effects of cross-gender hormones and gender-affirmation surgery on mental health). It is perhaps unsurprising that there is only a small collection of such interventions outlined in the peer-reviewed literature, as the Royal Australian and New Zealand College of Psychiatrists (2016) has previously highlighted that both gender and sexual minority people have historically been ‘criminalised, pathologised and invisibilised’ (para. 3) in their position statement which focused on recognizing and addressing the mental health needs of these under-served populations. These populations, however, should not be overlooked, especially when considering that according to population-based data, gender and sexual diversity are not that rare. In fact, in New Zealand, it is estimated that 9% of secondary school students are gender and sexual minority adolescents (Lucassen et al., 2019). This figure is inclusive of 1.2% of adolescents who identify as transgender and a further 2.5% who are not sure about their gender (Clark et al., 2014).

Socio-cultural contexts matter and typically gender minority adolescents (like other adolescents frequently exposed to mistreatment) cannot simply leave harmful social environments, due to the practical constraints around their schooling and their economic dependence on their families. Unfortunately a proportionately large percentage of transgender adolescents will have parents who mistreat them (Strauss et al., 2020b), many will also experience abuse from people outside their family (Strauss et al., 2020b) with bullying at school being particularly commonplace, especially when transgender adolescents are compared to cisgender adolescents (Clark et al., 2014). Hence, there is a need to address the ‘pervasive mistreatment and violence’ (James et al., 2016, p. 4) experienced by many trans individuals, which in turn is associated with compromised mental health. There is now also an urgent need for widely accessible and effective help to assist transgender adolescents to develop the best possible skills to thrive. Gender (and sexual) minority adolescents in high-income countries are thought to be ‘coming out’ earlier in relation to their gender and/or sexual minority status, further exacerbating the challenges (Gnan et al., 2019; Lucassen et al., 2015a). This trend is occurring even as transgender adolescents are acutely aware that the adults in their lives are instructing them to wait until they ‘grow up’ before ‘making decisions’ about their gender identity (Singh, 2013). This is noteworthy because an LGBT adolescent coming out before the age of 16 years is associated with compromised mental health, hypothesized to be related to peer group rejection and discrimination (Gnan et al., 2019). This suggests that interventions delivered when young people are still in school are likely to be required (Gnan et al., 2019). Assistance or interventions should be available online because prior work from Australia, the United Kingdom and North America has established that gender minority adolescents seek informal support online for issues pertaining to their mental health and well-being (Austin et al., 2020; McDermott et al., 2016; Singh, 2013; Strauss et al., 2017). Moreover, previous research has highlighted that professionals (including commissioners of mental health services) have recognized the importance of online interventions for widening access for the help designed to support these adolescents (Lucassen et al., 2018). However, the ‘Inverse Care Law’ refers to policies that (often inadvertently) restrict needs-based care in the populations with the poorest health outcomes (Watt, 2018) and that ‘the availability of good medical care tends to vary inversely with the need for it in the population served’ (Hart, 1971, p. 405). This is likely to apply to gender minority adolescents who have previously reported significant difficulties accessing health care (Clark et al., 2014). This is not necessarily because they are hard to reach, but because, like other under-served populations, they are easy to over-look or neglect (Watt, 2018).

Despite gender minority youth having very high mental health needs, and although when transgender youth are combined with those not sure about their gender they make up almost 4% of the adolescent population (Clark et al., 2014), some perceive that they are too small in size to warrant the roll-out of an LGBT- or transgender-specific online intervention (Lucassen et al., 2018). In theory such interventions can feasibly be provided across nations and regions in areas where there are no LGBT supportive mental health services when delivered as an electronic (digital) psychotherapy (e-therapy for brevity). Earlier work has already outlined how transgender-affirming adaptations can be made to cognitive behavioural therapy (CBT) (Austin and Craig, 2015) and research with other minority populations, specifically ethnic minorities, also suggests that therapy must be cognisant of a client’s culture and context (Miranda et al., 2005). Therapy attuned to a client’s context is understandably important in order to facilitate adequate engagement with treatment (Miranda et al., 2005). However, it has been suggested that a minority population-specific intervention may not actually be any more effective for minority individuals than an intervention designed for the majority (Pachankis, 2018).

SPARX is a form of self-help computerized CBT for adolescents with symptoms of depression, shown to be least as good as treatment as usual in primary healthcare settings (Merry et al., 2012). Since April 2014 SPARX has been made freely available online to everyone with a New Zealand IP address (see www.sparx.org.nz). Although the original version of SPARX was developed with input from LGBT young people (Lucassen et al., 2013) and a ‘rainbow version’ was developed and assessed in a mixed methods open trial (Lucassen et al., 2015b, 2015c), only the original ‘mainstream’ version of the program is currently available in New Zealand. Very few e-therapies have been made available to adolescents on a national level and to the best of our knowledge none have gathered data on gender minority users. The aim of this study was to explore whether a ‘mainstream’ resource like SPARX, which was developed to appeal to a diverse range of adolescents, can support the mental health of transgender adolescents as well as it supports the mental health of cisgender adolescents. We developed three a priori hypotheses for this research. First, given that transgender adolescents are more likely to report clinically significant depressive symptoms in comparison with their cisgender peers, we hypothesized that transgender adolescents registering to use SPARX would also be more likely to have high mental health needs at baseline relative to both male and female adolescent registrants. Second, as individuals in the community with high mental health needs may engage more fully with e-therapies (March et al., 2018), we hypothesized that transgender adolescents would be more likely to complete SPARX, than both male and female registrants. Finally, we hypothesized that transgender adolescents would have equivalent reductions in depressive symptoms when compared to both male and female registrants, as the SPARX program was created to support the mental health of a wide cross section of adolescents.

## Methods

We conducted a secondary analysis of 5 years of SPARX usage data, from the national launch in New Zealand (i.e. from April 2014). Thus, this study is a large open ‘real world’ trial of SPARX among male, female and transgender users of the program. Over that period the program was actively promoted via social media advertising (specifically on Facebook), government websites (e.g. by the New Zealand Ministry of Health https://www.health.govt.nz/your-health/services-and-support/health-care-services/mental-health-services/mental-health-services-where-get-help), schools and health professionals. At registration individuals are asked to respond to a combined sex/gender question, where a person could select only one of four options, in particular ‘Male’, ‘Female’, ‘Transgender’ or ‘Intersex’ (of note, the results pertaining to intersex users of SPARX are to be reported elsewhere). Individuals were also asked their ethnicity (where they could select more than one response). To be included in the analyses participants:

- Had to be within the SPARX intervention’s target age range at registration (i.e. 12–19 years old);
- Had to identify as male, female or transgender;
- Had to have completed the program’s registration process and begun using the SPARX program (i.e. had started Module 1).

All participants in this study had therefore, at the very least, begun the SPARX program which is described briefly below.

### Ethics

The study was approved by the New Zealand Health and Disability Ethics Committee (HDEC) Reference: 15/NTB/183 (Youth e-Therapy Implementation Study).

### The intervention

**Figure 1.**
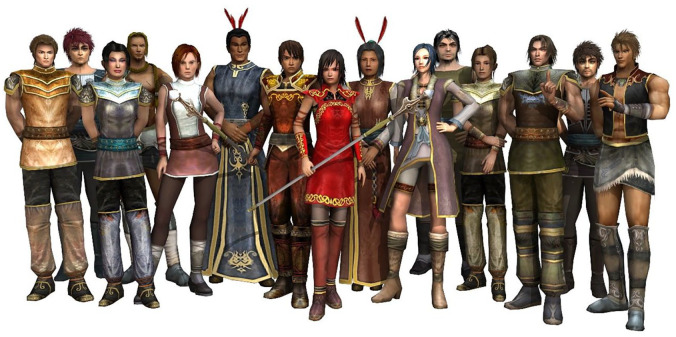
SPARX characters (the female character dressed in red holding a staff and the male character beside her also holding a staff are the avatars).

SPARX is a computerized CBT self-help program for the treatment of depressive symptoms in adolescents. It uses the medium of a fantasy world, where the user’s avatar is faced with a series of challenges to rid a virtual world of gloom and negativity (Merry et al., 2012). Each of the seven modules (or levels) takes approximately 30 minutes to complete and each has a direct teaching component whereby a skill from the fantasy world is applied to the user’s real-life context (Merry et al., 2012). It has been rolled out nationally in New Zealand with the Patient Health Questionnaire - modified for Adolescents (PHQ-A) integrated into the program. The PHQ-A (further outlined in the ‘Outcome Measure’ section below) replaced a Likert-type-based mood scale used in earlier clinical trials of the program with the PHQ-A embedded in Modules 1, 3 and 7 of SPARX for the purposes of the national roll-out. Every module begins with ‘the Guide’ (a type of ‘virtual therapist’) greeting the user, providing information about depression and, after the first module, reviewing the content covered in the last module a user completed. Each user customizes their avatar (see Figure 1), which, in SPARX, consists of a character that is ‘conventionally’ male or female in appearance (i.e. the avatar options are gender binary). In each module the user enters the fantasy world and their avatar completes a mission, after which ‘the Guide’ explains the significance of the challenge and how it applies to real life. SPARX is based on core CBT skills, and set challenges (i.e. homework tasks) are provided to allow practice and facilitate skill generalization. The main CBT skills covered in SPARX are physically represented by ‘the shield against depression’ with six ‘gem stones’ each of which corresponds to certain CBT content, specifically: ‘Relax’ (relaxation training), ‘Do it’ (behavioural activation), ‘Sort it’ (social skills training), ‘Spot it’ (recognizing or naming cognitive distortions), ‘Solve it’ (problem-solving) and ‘Swap it’ (cognitive restructuring). The shield has a central core, which is hope. The shield against depression is used at key stages throughout SPARX to highlight the skills used, and the user collects a ‘gem stone’ associated with the shield (gems which they find in the fantasy world during the course of each module) as a reward for completing that level. The physical appearance of each module is reflected in metaphors for the concepts covered. For instance, Module 4 is the ‘Mountain Province’ and users are required to apply problem-solving (‘Solve it’) skills in order to climb a mountain in that level.

### Outcome measure

The PHQ-A is a brief severity measure of depressive symptoms for adolescents, it is frequently used in primary healthcare and has sound psychometric properties (Johnson et al., 2002). Specifically, it has previously demonstrated satisfactory sensitivity (73%), specificity (94%) and overall diagnostic accuracy (92%) (*n* = 403) when compared to clinical interview for major depressive disorder in adolescents (Johnson et al., 2002). It consists of nine items, focused on the past 2 weeks in this study, based on the lead question ‘How often have you been bothered by each of the following symptoms …?’ measured on a 4-point scale (0 = Not at all; 1 = Several days; 2 = More than half the days; and 3 = Nearly every day). The last question, which assesses an adolescent’s thoughts of suicide and self-harm, is ‘Thoughts that you would be better off dead, or of hurting yourself in some way?’ Total scores range from 0 to 27, and depression symptom severity is categorized: 0–4 = No or minimal symptoms; 5–9 = Mild depression; 10–14 = Moderate depression; 15–19 = Moderately severe depression; and 20–27 = Severe depression. In order to assess for changes in depressive symptoms SPARX users are asked to complete the PHQ-A at the start of Module 1 (i.e. baseline), at Module 4 (i.e. a mid-point assessment) and at Module 7 (i.e. the post-SPARX measure).

### Analyses

Descriptive statistics including numbers, percentages, means and standard deviations were used to summarize results related to participant demographics, baseline depression, SPARX adherence and engagement and changes in PHQ-A scores. In relation to ethnicity, participants who chose more than one ethnic group were assigned a single ethnic group using using a Statistics New Zealand (2002) ethnicity prioritization method. Adolescents were categorized as having ‘high mental health needs’, if they had a baseline PHQ-A total score in the ‘moderately severe depression’ or ‘severe depression’ range and/or a baseline self-harm and suicidal ideation score indicating medium or above risk. Specifically, in this study a response of 2 (i.e. ‘More than half the days’) or 3 (i.e. ‘Nearly every day’) for the PHQ-A suicide and self-harm item. Chi-square tests for independence were used to compare transgender participants to male and female participants for categorical data. For continuous data (e.g. baseline PHQ-A scores), means for transgender, male and female participants were compared with a one-way between-groups analysis of variance (ANOVA). Statistical analyses were performed using IBM SPSS Statistics version 24. A *p* value of <0.05 was taken to indicate statistical significance in all analyses.

## Results

There were 13,489 youth within the target age range (i.e. 12–19 years old) who had accessed SPARX, primarily via a personal computer, although more recently users have been able to access SPARX on electronic devices (e.g. tablets) or their mobile phone. In total, 9079 youth completed the registration process and began using the SPARX program (i.e. they had at least begun Module 1). Of those completing registration and using SPARX, approximately two-thirds reported that they were female (65.7%), almost one-third reported that they were male (32.0%) and 2.3% reported that they were transgender. The majority of users were younger adolescents, who were aged between 12 and 15 years old (Table 1). However, male users were more likely to be younger than female and transgender users (*p* < 0.001). Broadly reflective of the overall adolescent population in New Zealand, approximately two-thirds of SPARX users were New Zealand European, with the next largest ethnic group of users being Māori. There were no significant differences by gender group and ethnicity.

**Table 1.** Demographics.

	Transgender (*N* = 294)	Male (*N* = 4135)	Female (*N* = 9060)	Statistical comparison
Age (years)				
12–15	131 (63.3%)	1927 (66.4%)	3595 (60.2%)	χ^2^(2) = 31.22, *p* < 0.001
16–19	76 (36.7%)	977 (33.6%)	2373 (39.8%)	
Ethnicity^a,b^				
Māori	32 (15.5%)	458 (15.8%)	881 (14.8%)	χ^2^(8) = 10.85, *p* = 0.21
Pacific	9 (4.3%)	103 (3.5%)	221 (3.7%)	
Asian	8 (3.9%)	231 (8.0%)	466 (7.8%)	
Other	21 (10.1%)	236 (8.1%)	432 (7.2%)	
NZ European	137 (66.2%)	1876 (64.6%)	3968 (66.5%)	

a Prioritized ethnicity – if multiple ethnicities were selected users were assigned a single ethnic group (based on a Statistics NZ ethnicity prioritization method) in the following order: Māori; Pacific; Asian; ‘Other’ ethnic groups (except NZ European); NZ European.

b Missing ethnicity data for three females and one male participant.

More than 9 out of 10 (91.4%) of those completing registration and beginning SPARX completed the baseline depression assessment. Of the transgender users completing the baseline depression assessment (*n* = 185), more than two-thirds were categorized as having high mental health needs (69.0%) and they were significantly more likely to have high needs in comparison with males and females, χ^2^(2) = 289.79, *p* < 0.001. Transgender and female SPARX users had higher mean PHQ-A scores than males, with the mean scores of the male users of SPARX in the moderate depression range (*M* = 11.1), while mean scores for the transgender and female users of the program were in the moderately severe depression range (at *M* = 16.7 and 14.7, respectively) (Table 2). A one-way between-groups ANOVA comparing transgender, male and female SPARX users in terms of mean PHQ-A score at baseline was conducted, and the differences were significant, *F*(1, 192.36) = 38.68 *p* < 0.001.

**Table 2.** Baseline depression.

Baseline PHQ-A	Transgender (*n* = 185)	Male (*n* = 2621)	Female (*n* = 5489)
No or minimal symptoms (scores < 5)	24 (13.0%)	644 (24.6%)	572 (10.4%)
Mild depression (scores 5–9)	10 (5.4%)	566 (21.6%)	822 (15.0%)
Moderate depression (scores 10–14)	30 (16.2%)	548 (20.9%)	1165 (21.2%)
Moderately severe depression (scores 15–19)	38 (20.5%)	417 (15.9%)	1300 (23.7%)
Severe depression (scores ⩾ 20)	83 (44.9%)	446 (17.0%)	1630 (29.7%)
High mental health needs	129 (69.0%)	978 (37.3%)	3109 (56.6%)
	Mean (SD)	Mean (SD)	Mean (SD)
Mean PHQ-A score	16.7 (8.2)	11.1 (7.8)	14.7 (7.2)

PHQ-A: Patient Health Questionnaire-modified for Adolescents; SD: standard deviation.

Approximately half of those completing registration and beginning SPARX completed at least the first module (i.e. they were well orientated to the program with males being less likely to complete a module compared to females and transgender adolescents (*p* < 0.038) (Table 3). Transgender users who had completed at least Module 1 of SPARX were less likely to complete Module 4 (*p* = 0.005) and Module 7 (*p* = 0.048) in comparison with males and females who had completed at least Module 1. However, overall completion rates at Modules 4 and 7 were low for all users of the program (i.e. less than 10%), and numbers were particularly small for transgender users at both Module 4 and Module 7. Users of SPARX (on average) spent less than 25 minutes per module of SPARX in this study. There were no significant differences between the three groups on average time spent per module, *F*(1112.74) = 0.026, *p* = 0.99.

**Table 3.** SPARX adherence and engagement.

SPARX module completions	Transgender (*n* = 207)	Male (*n* = 2904)	Female (*n* = 5968)	Statistical comparison
At least Module 1	111 (53.6%)	1504 (51.8%)	3263 (54.7%)	χ^2^(2) = 6.54, *p* = 0.038
Between Modules 1 and 3	99 (47.8%)	1283 (44.2%)	2692 (45.1%)	χ^2^(4) = 14.99, *p* = 0.005
Module 4 or more	12 (5.8%)	221 (7.6%)	571 (9.6%)	
Between Modules 1 and 6	106 (51.2%)	1411 (48.6%)	3024 (50.7%)	χ^2^(4) = 9.61, *p* = 0.048
Completed all 7 modules	5 (2.4%)	93 (3.2%)	239 (4.0%)	
	Minutes (SD)	Minutes (SD)	Minutes (SD)	
Average time spent (in minutes) per module	23.5 (36.3)	21.4 (21.5)	25.4 (30.2)	*F*(1112.74) = 0.03, *p* = 0.99

SD: standard deviation.

Of those completing a baseline and subsequent PHQ-A, both male and female users of SPARX had improvements in their scores (mean improvement of 2.68 and 3.15 points, respectively), whereas transgender users did not. The changes in PHQ-A scores were statistically significant with both males and females improving and transgender users showing no improvement (Table 4). However, caution is required when interpreting these results, given the very small number of transgender users (i.e. *n* = 14).

**Table 4.** Changes in PHQ-A scores.

Change in PHQ-A total score from baseline to last Module (4 or 7)	Improvements in PHQ-A scores					Statistical comparison
	*N*	Change in score		95% CI for mean		
		Mean	SD	Lower CI	Upper CI	
Transgender	14	–0.43	11.09	–6.83	5.97	*F*(1888) = 3.93, *p* = 0.048
Male	247	2.68	6.68	1.84	3.52	
Female	630	3.15	5.92	2.68	3.61	

PHQ-A: Patient Health Questionnaire-modified for Adolescents; CI: confidence interval; SD: standard deviation.

## Discussion

### Principal findings and comparisons to previous research

This is the first study describing the impact of an e-therapy on transgender young people. Several points stand out. The first is that 2.3% of SPARX registrants reported that they were transgender, which is almost double the proportion of the overall transgender adolescent population (i.e. 1.2%) previously estimated in New Zealand (Clark et al., 2014). This suggests that transgender young people will use an e-therapy, such as SPARX.

A second key point is that, as hypothesized, transgender registrants had high mental health needs, with more than two-thirds of these youth being categorized as such. Furthermore, almost half (i.e. 44.9%) had a PHQ-A baseline score in the ‘severe depression’ range. This is in line with the statement from the Royal Australian and New Zealand College of Psychiatrists (2016) that greater attention is urgently needed to address the high vulnerability and poor mental health outcomes of gender minority individuals.

A third key point is that completion rates in this study were very low compared to trial data, with less than 10% of participants completing Module 4 and less than 4% completing Module 7, compared with 86% and 60%, respectively, in the original SPARX study (Merry et al., 2012). Our completion rates in this study are in line with experiences from other implementation efforts, with adherence to e-therapy interventions one of the major challenges faced currently (Graham et al., 2019). It has been estimated that e-therapy engagement rates are 4.06 times higher in published trials (where participants are proactively recruited and followed up) in comparison with the real-world usage of the exact same program (Baumel et al., 2019). Furthermore, a systematic review of the real-world uptake and engagement of digital self-help interventions reported that only 0.5% to 28.6% of users complete interventions in the ‘real world’ (Fleming et al., 2018). However, SPARX completion rates for transgender users, most of whom had high mental health needs, were especially disappointing, especially in comparison with male and female users.

A fourth key point is that the ‘look and feel’ of SPARX for gender minority adolescents may in part explain the low completion rates. The forced gender binary is inherent in the program (i.e. the user can only customize a male or female avatar with no in-between option). This is an issue LGBT youth have identified, and they have recommended non-binary options for gender diverse users of the program (Lucassen et al., 2018). Other aspects of SPARX may also be problematic for transgender young people, such as the lack of representation of gender diverse characters that are ‘out’ (i.e. are open about being a gender minority individual). However, earlier work suggests that while some trans adolescents will not want to access interventions specifically targeted towards LGBT youth, others have recommended programs be specifically targeted and tailored to meet the needs of gender minority youth (Strauss et al., 2019). It is not yet known whether e-therapies designed specifically to treat depression in transgender adolescents will yield better results than a ‘mainstream’ resource like SPARX in its current form. Efforts are underway to refine SPARX in Australia, where the intervention is being adapted specifically for transgender young people. This version of SPARX will soon be evaluated among transgender adolescents in Western Australia (Strauss et al., 2019). Furthermore, an evaluation of AFFIRM, a bespoke transgender affirmative CBT intervention delivered face-to-face in Canada, was effective in significantly decreasing depression scores in a pilot study with eight transgender adolescents where decreases persisted through to a 3-month follow-up assessment (Austin et al., 2018) indicating the preliminary efficacy of a tailored depression intervention for transgender young people. Moreover, seven out of the eight transgender participants in the study either strongly agreed or agreed that they ‘would recommend AFFIRM to other youth with sexual and/or gender minority identities’ (Austin et al., 2018, p. 6).

The fifth and final key point is that, on average, transgender users did not report improvements in their depressive symptoms, although this is a tentative finding given the very small number of transgender users completing more than one PHQ-A assessment (i.e. *n* = 14). Nevertheless, the high demonstrated need, poor SPARX completion rates relative to males and females, and our tentative results in relation to depression scores indicate more should be done to support the mental health of transgender adolescents.

### Strengths

This study has several strengths. In particular, to the best of our knowledge, it is the first national study of transgender adolescents seeking assistance for their mental health utilizing an e-therapy. The measure built in (i.e. the PHQ-A) is widely used, psychometrically sound and was completed by all users who engaged with the applicable modules. The large-scale ‘real-world’ context of this study is a strength, as the participants used a freely available self-help intervention. Because there were almost 10,000 users included in this study, a sizable number of transgender users were included, and despite only 14 transgender participants completing more than one PHQ-A assessment, the study is comparable in size to other treatment studies in the trans youth field (e.g. Austin et al., 2018).

### Limitations

Although almost 300 transgender adolescents were originally recruited, completion rates were disappointingly low. Demographic data and symptom scores were based on self-report. The single sex/gender item was restrictive, because users could only select ‘Male’, ‘Female’, ‘Transgender’ or ‘Intersex’ and they were not permitted to select more than one response, nor where they given the opportunity to describe their gender in their own words. Ideally gender identity should have been determined using the two-item approach, as recommended by transgender health experts (e.g. Reisner et al., 2014), specifically an item on sex assigned at birth followed by an item on gender identity. Employing this two-item response may have resulted in an even larger proportion of gender minority participants than in our study. This is especially true when the second item is inclusive, as when Rider and colleagues asked participants, ‘Do you consider yourself transgender, genderqueer, genderfluid, or unsure about your gender identity?’ (response options = yes or no) among over 80,000 adolescents in the Minnesota Student Survey, 2.7% were categorized as transgender and gender nonconforming (Rider et al., 2018). Striking a balance between routine collection of data, which is concise and comprehensible for all adolescents, that does not adversely affect registration alongside gathering more detailed data for unique sub-populations creates some challenges. These challenges could be overcome with specific transgender interventions (either online or offline), as in the AFFIRM intervention, where psychotherapies such as CBT are refined with gender minority adolescents in mind. Alongside the need to develop evidence-based interventions that support the mental health of trans adolescents are improvements in regard to system-level policies in education, health care and community settings (Coyne et al., 2020). Policies that reduce trans adolescents’ exposure to harmful and distressing experiences are necessary, as bullying and discrimination have repeatedly been highlighted as negatively impacting upon the mental health of trans adolescents (Coyne et al., 2020).

## Conclusion

Adolescent transgender users of the SPARX program had high mental health needs. The poor completion rates and lack of change in depressive scores indicate that SPARX is probably less effective for these young people. SPARX, as currently delivered without any adaptations, does not appear to meet the needs of this group. More must be done to address the mental health problems faced by transgender adolescents and this study underscores the need to design or refine interventions so that they appeal to gender minority youth.
